# Rapamycin for aging stem cells

**DOI:** 10.18632/aging.103816

**Published:** 2020-08-04

**Authors:** William S. Hambright, Marc J. Philippon, Johnny Huard

**Affiliations:** 1 Steadman Philippon Research Institute, Vail, CO 81657, USA; 2 The Steadman Clinic, Vail, CO 81657, USA

**Keywords:** rapamycin, aging, stem cells, progeria

Aging is generally defined as a time-dependent decline in physiological function and reserve [1]. Hallmarks of aging include cellular senescence, stem cell exhaustion, DNA damage, telomere attrition, and deregulated nutrient sensing among others [1]. Age-associated stem cell dysfunction has been well characterized in various tissue specific stem cell populations [2] leading to a decline in regenerative potential of tissues. Some stem cell populations have been found to increase in number with age (hematopoietic stem cells for example), while many others such as neural stem cells and muscle progenitor cells deplete with age whereby stem cell pools become exhausted leading to reduced regeneration/repair capacity of tissue [1,2]. However, stem cell dysfunction due to cell intrinsic factors (DNA damage, oxidative stress, and mitochondrial dysfunction) along with extrinsic factors (chronic inflammation, changes in niche) is a hallmark of aging. It turns out that mTOR (mammalian target of rapamycin) plays a significant role in stem cell dysfunction through various mechanisms highlighting its potential as an anti-aging target to rejuvenate stem cell function. In fact, mTOR regulates many of the hallmarks of aging. A breakthrough study in 2009 by Harrison et al. showing the lifespan extending properties of rapamycin in genetically heterogenous mice led to significant study of rapamycin as an anti-aging intervention. Since that time, rapamycin has been well studied in aging and age-related functional decline mainly through the modulation of autophagy, mitochondrial function, insulin signaling, and senescence [3].

TOR is a heavily conserved serine/threonine kinase with homologues in several eukaryotes from yeast to humans highlighting its importance in cellular processes [3]. The mammalian version, mTOR exists as two distinct complexes, mTOR1 and mTOR2 that are structurally and functionally different [3,4]. The mTOR1 complex acts as a central nutrient sensor and regulator of proliferation, growth, and survival [3,4]. Rapamycin is a potent natural inhibitor of mTOR through binding of FK506-binding proteins thus destabilizing mTORC1 primarily, but also to some degree, can prevent phosphorylation downstream mTOR1 targets. mTOR2 activity is usually preserved during acute Rapamycin treatment but prolonged exposure can reduce mTOR2 activity as well [3,4]. While mTOR is critical for anabolism and proliferation, including in certain stem cell populations, its aberrant activation has been linked to stem cell dysfunction during aging by our group and others [2,3,5,6]. It has been shown that hematopoietic stem cells (HSC) from naturally aged mice have elevated mTOR1 activity and increasing mTOR1 signaling in young mice induces premature aging [3]. In progeroid (accelerated aging) murine models, mTOR is increased in various progenitor cells as well. In the *Ercc1^-/Δ^* progeria mouse model, rapamycin improves muscle-derived stem cell (MDSC) function through induction of autophagy [6]. Genetic ablation of the downstream mTOR target S6K also results in increased lifespan and preserved HSC function [3]. Recently, we reported that Rapamycin treated primary MDSCs from the *Zmpste24^-/-^* progeria mouse model could restore differentiation and proliferation potential, reduce senescence, and enhance autophagy [5]. Thus, hyperactive mTOR activity with aging seems to have deleterious consequences in somatic stem cells, especially MDSCs.

The data suggesting rapamycin as an anti-aging intervention is compelling not only for natural aging but also progeroid conditions. Along these lines, rapamycin has been found to delay senescence, reduce nuclear membrane deformation, and stimulate degradation of progerin in fibroblasts from patients with Hutchinson-Gilford Progeria Syndrome (HGPS). Moreover, activation of the autophagic pathway using rapamycin can counteract progerin and farnesylated prelamin A accumulation in HGPS cells. In our lab, we have also found that rapamycin treatment can rescue nuclear membrane deformation in MDSCs derived from the *Zmpste24^-/-^* HGPS model. Thus, rapamycin can partially attenuate defective lamin A processing, potentially in somatic stem cells, a process previously not linked to rapamycin treatment***.*** Indeed, at least one clinical trial for HGPS involves a rapamycin derivative (everolimus) as a co-therapy (NCT02579044).

Finally, rapamycin and other compounds have been demonstrated to have significant senotherapeutic effects (i.e. selective ability to restore or eliminate senescent cells) [5,6]. Many senotherapeutic drugs have minimal side effects and are effective with transient dosing. For example, at our clinic we routinely study the senotherapeutic drug fisetin, a phytonutrient with potent senolytic ability, and are conducing multiple randomized trials for its use in the context of osteoarthritis, an age-related pathology. Not only has rapamycin has been demonstrated to reduce senescence in MDSCs by our group [5], but others have demonstrated that blocking mTOR reduces stem cell senescence and associated secretory phenotypes [3,7]. In orthobiologic applications, the use of senotherapies like rapamycin is very compelling to reduce the anti-regenerative senescent profile of autologous stem cell therapies like bone marrow concentrate in various orthopedic indications.

Should rapamycin be prescribed ubiquitously as an anti-aging supplement? There is certainly a preponderance of evidence demonstrating the safety of rapamycin in healthy and aged humans that has been well reviewed [8]. Since its approval in 1999 by the FDA, rapamycin has been used by millions of patients with very few mild but reversible side effects. However, one possible strategy is likely intermittent treatment at higher doses for prolonged periods of time. We additionally propose that a combinatorial approach may be in order to target senescence at multiple nodes (inhibition of anti-apoptotic pathways and mTOR) directly through the use of multiple senotherapeutic agents such as fisetin and rapamycin. Overall, the plethora of preclinical and clinical data using rapamycin strongly suggests that targeting mTOR and/or senescence is a promising therapeutic strategy to mitigate aging-related phenotypes and restore stem cell health and function.

## References

[1] López-Otín C, et al. Cell. 2013; 153:1194–217. 10.1016/j.cell.2013.05.039 23746838 PMC3836174

[2] Meng D, et al. Development. 2018; 145:dev152595. 10.1242/dev.152595 29311260 PMC5825873

[3] Papadopoli D, et al. F1000 Res. 2019; 8:8. 10.12688/f1000research.17196.1 31316753 PMC6611156

[4] Hausch F, et al. Cell Cycle. 2013; 12:2366–70. 10.4161/cc.25508 23839048 PMC3841315

[5] Kawakami Y, et al. Mol Ther Methods Clin Dev. 2019; 14:64–76. 10.1016/j.omtm.2019.05.011 31312666 PMC6610712

[6] Takayama K, et al. J Orthop Res. 2017; 35:1375–82. 10.1002/jor.23409 27572850 PMC5516198

[7] Iglesias-Bartolome R, et al. Cell Stem Cell. 2012; 11:401–14. 10.1016/j.stem.2012.06.007 22958932 PMC3477550

[8] Blagosklonny MV. Aging (Albany NY). 2019; 11:8048–67. 10.18632/aging.102355 31586989 PMC6814615

